# Effects of a Structured Recreational Activity Program on Self-Regulation in Preschool Children

**DOI:** 10.3390/children13101321

**Published:** 2026-09-28

**Authors:** Gaye Erkmen Hadi, Özlem Yalçın Kişi, Ezgi Ertüzün, Koulla Parpa

**Affiliations:** 1 Recreation Department, Faculty of Sport Sciences, Selçuk University, Konya 42100, Turkey; ezgiulker@selcuk.edu.tr; 2 Sport and Exercise Science, University of Central Lancashire Cyprus, Larnaca 7080, Cyprus; kparpa@uclan.ac.uk

**Keywords:** preschool children, self-regulation, recreational activity program, structured play, early childhood

## Abstract

**Highlights:**

**What are the main findings?**

• Participation in a 12-week structured recreational activity program was associated with greater short-term improvements in self-regulation among preschool children.
• Patterns of change over time favored the RAP group across the assessed self-regulation outcomes.

**What are the implications of the main findings?**

• Structured recreational activities may support behavioral self-regulation and positive emotional engagement.
• The findings support further evaluation of RAP in preschool settings.

**Abstract:**

**Background/Objectives:** This study examined changes in self-regulation among preschool children participating in a structured Recreational Activity Program (RAP) compared with children receiving routine preschool education. **Methods:** The study employed a pretest–posttest control group design with classroom-level random allocation. Two existing classrooms from one participating preschool were randomly allocated to the RAP or control condition. The primary analysis included 38 children aged 5–6 years (RAP: *n* = 19; control: *n* = 19). Self-regulation was assessed using the Preschool Self-Regulation Assessment (PSRA). The total self-regulation score was the primary outcome, while Attention/Impulse Control and Positive Emotion were secondary outcomes. The RAP was delivered over 12 weeks (24 sessions) and integrated rhythm- and movement-based activities, structured games, and relaxation-oriented practices. Changes over time were evaluated using nonparametric longitudinal analysis, with the Group × Time interaction as the effect of interest. Between-group differences in individual change scores and 95% bootstrap confidence intervals were examined as supporting analyses. **Results:** Significant Group × Time interactions were observed for total self-regulation (WTS = 53.47, *p* < 0.001), Attention/Impulse Control (WTS = 42.03, *p* < 0.001), and Positive Emotion (WTS = 50.28, *p* < 0.001), indicating different patterns of change over time between the RAP and control groups. Supporting change-score analyses showed greater observed increases in the RAP group for total self-regulation (mean difference = 14.26, 95% CI [11.37, 17.21]), Attention/Impulse Control (mean difference = 7.68, 95% CI [5.16, 10.26]), and Positive Emotion (mean difference = 6.53, 95% CI [4.89, 8.05]). A baseline difference in Attention/Impulse Control was present between the groups and should be considered when interpreting this outcome. **Conclusions:** Participation in the RAP was associated with greater short-term improvements in the assessed self-regulation outcomes compared with routine preschool education. However, because only one classroom was allocated to each condition, potential intervention effects cannot be separated from classroom- or teacher-level influences. The findings should therefore be regarded as preliminary and warrant confirmation in studies involving multiple independent classrooms within each condition. Trial registration: ClinicalTrials.gov, NCT06360016; retrospectively registered on 10 April 2024.

## 1. Introduction

Early childhood is widely recognized as a crucial developmental period in which cognitive, behavioral, and emotional capacities develop and are structured rapidly [1]. Among these abilities, self-regulation is regarded as a significant indicator of children’s later academic success, psychological well-being, and social functioning. A substantial body of research further indicates that self-regulation skills have long-term effects across all developmental areas in early childhood and play a central role in shaping developmental trajectory [2,3,4,5,6]. At the same time, early childhood is a period in which children increasingly engage in play and recreational experiences that shape how they interact with their environment, respond to social expectations, and regulate their behavior. When used constructively, such experiences may provide a meaningful context for the development of executive functioning and self-regulation through repeated engagement, enjoyment, and social interaction [7,8,9,10,11,12,13]. While leisure is often conceptualized as relatively unstructured free-time activity, the present study adopts a structured recreational approach designed to intentionally support self-regulatory processes through guided and developmentally appropriate activities. Accordingly, rather than equating leisure experiences with structured interventions, this study draws on leisure and play-based perspectives to inform the design of a structured recreational context aimed at supporting self-regulation in early childhood.

Leisure-time experiences may exert both immediate and cumulative developmental effects, influencing individuals’ momentary psychological states while also contributing to longer-term developmental trajectories through repeated engagement over time [14,15,16]. Accordingly, unlike formal academic settings, such environments provide intrinsically motivating and relatively low-pressure contexts that allow children to actively engage with and reflect on their experiences, thereby supporting developmental processes associated with behavioral and emotional regulation [17].

From a developmental perspective, self-regulation represents a central developmental competency supported within such contexts. In the present study, self-regulation is conceptualized as children’s capacity to control attention, manage impulses, and regulate emotional responses in order to behave adaptively in goal-directed situations [18]. In early childhood studies, these skills are assessed through observable behaviors such as attention control, impulse regulation, and emotionally appropriate participation in structured activities [3,19,20]. Accordingly, the present study focuses specifically on these behavioral and emotional components of self-regulation, rather than broader developmental dimensions such as long-term goal setting or motivational regulation. This focused conceptualization is particularly appropriate for preschool populations, in which self-regulatory capacities are still emerging and highly responsive to structured environmental experiences. Longitudinal evidence further indicates that behavioral self-regulation develops rapidly between the ages of three and seven, although children may follow different developmental trajectories depending on individual and contextual characteristics [21]. Indeed, self-regulation develops rapidly during the preschool years and constitutes a foundational developmental competency that continues to mature throughout childhood and adolescence [22]. Beyond its role in supporting cognitive and behavioral functioning, self-regulation has also been associated with broader developmental outcomes, including physical health, resilience, and adaptive functioning across diverse childhood contexts. Previous research suggests that stronger self-regulatory capacities may function as a protective factor, particularly for children exposed to adversity or environmental stress [23]. Together, these findings highlight the importance of interventions designed to strengthen self-regulation during the preschool years.

Children with stronger self-regulation skills are known to be more successful at maintaining attention, controlling impulses, and managing emotional responses, which facilitates both learning and social adaptation [24,25,26]. However, self-regulation does not develop in isolation; rather, it emerges within interpersonal and environmental contexts through children’s interactions with caregivers and their broader social and activity settings. Recent developmental perspectives further emphasize that emotional development and regulation are shaped through relational processes, including parent–child interactions and co-regulatory experiences, as well as through the broader environmental contexts in which these interactions occur [27]. In this context, early childhood environments that provide opportunities for active participation, structured interaction, and emotionally meaningful involvement can play a central role in supporting attention, behavior, and emotional regulation, and thus in the development of self-regulation skills [28,29].

In this regard, play and recreational activities are widely recognized as developmentally appropriate environments in which children can practice behavioral control, emotional expression, and social cooperation skills [30,31,32,33]. Through collaborative play and structured group activities, children learn to follow rules, regulate their impulses, negotiate with peers, and respond to social expectations. Recreational activity contexts can further support these processes by providing enjoyable and intrinsically motivating experiences that encourage sustained participation.

Furthermore, studies suggest that structured play and movement-based interventions applied in such contexts may support the development of self-regulation skills and executive functions in early childhood [34,35]. Based on this body of evidence, structured recreation programs incorporating play-based activities may offer valuable opportunities to support self-regulation development by providing organized, socially interactive, and developmentally meaningful contexts that encourage attention control and behavioral regulation [30,36,37,38,39,40,41].

Rule-based games and activities that combine movement experiences and collaborative interactions may support children’s ability to maintain attention, prevent impulsive responses, and interact constructively with their peers [37,42]. When designed to be both enjoyable and developmentally appropriate, such activities may also foster intrinsic motivation, persistence, and goal-directed behavior, which are closely linked to self-regulatory functioning [43,44,45]. In addition, relaxation-oriented practices, such as basic yoga and meditation, may further contribute to self-regulatory processes by supporting arousal regulation, sustained attention, and behavioral control during structured tasks [46,47,48].

Empirical studies provide further support for the effectiveness of play-based interventions in promoting self-regulation during early childhood. For example, the Red Light, Purple Light intervention program has been shown to improve behavioral self-regulation and early academic skills through structured group-based activities [49]. Previous research has shown that participation in recess games is associated with higher levels of self-regulation and earlier academic performance in preschool children [50]. Other research indicates that play-based physical activities and thematic play interventions can enhance motivation, social adaptation, and behavioral regulation [51,52]. Collectively, these findings are consistent with recent evidence syntheses suggesting potential benefits of multicomponent, developmentally appropriate approaches for supporting preschool children’s self-regulation [53,54].

Intervention approaches developed in recent years increasingly integrate movement, rhythm, and structured activity forms to support self-regulation skills in early childhood. For example, the Rhythm and Movement for Self-Regulation (RAMSR) program incorporates music- and rhythm-based activities to support executive functioning and self-regulation in preschool children, with positive findings reported across randomized controlled trials conducted in Australia and Hong Kong [55,56]. Similarly, the Kids’ Skills intervention program aims to support the development of self-regulation capacity through rule-based tasks, collaborative problem-solving processes, and structured interactions [57]. Recent systematic reviews further support the potential value of developmentally appropriate interventions incorporating play-, movement-, and mindfulness-oriented components [53,54,58]. However, programs that integrate these complementary elements within a unified recreational framework remain comparatively limited.

Self-regulation, however, involves the coordinated functioning of attention, behavior, and emotional processes. From this perspective, interventions that combine different types of experiences may provide broader opportunities for children to practice self-regulatory skills. Within this integrated framework, rule-based games may provide opportunities to practice inhibitory control and rule following; rhythmic and movement-based activities may support sustained attention, behavioral coordination, and engagement; socially interactive activities may provide opportunities to practice cooperation and behavioral adjustment in response to others; and relaxation-oriented practices may support arousal regulation and attentional control. Rather than treating these components as independent interventions, the RAP integrates them within a structured recreational context in which complementary self-regulatory processes can be practiced repeatedly across activities. Thus, the combination of game-based interaction, rhythmic movement, relaxation-oriented practices, and social interaction may provide a context for practicing attentional control, behavioral regulation, and positive emotional engagement. However, few experimental studies have evaluated recreational activity programs that integrate these complementary components within a single structured intervention for preschool children. While attention and behavioral processes are frequently emphasized, positive emotional engagement that emerges during structured activities is also recognized as an important component of self-regulation in early childhood. In early childhood research, positive emotion is generally addressed through observable affective responses that children exhibit during activities. These responses are defined by indicators such as enjoyment of the activity, willingness, motivation to participate, and displaying a positive and cooperative attitude during the task process [20,59]. These types of affective responses can indicate the extent to which children are emotionally involved in activities and how ready they are to engage in goal-oriented behaviors. Within the scope of the Preschool Self-Regulation Assessment, these behaviors are evaluated based on observations made during structured tasks requiring positive emotion, attention, and behavioral control. From a theoretical perspective, positive emotional engagement is considered to be closely related to self-regulation processes. According to the broaden-and-build theory, positive affect broadens the scope of attention and increases cognitive flexibility, supporting the exhibition of adaptive behaviors and the maintenance of sustained activity [60]. In early childhood contexts, environments that promote enjoyment, social interaction, and active participation may therefore facilitate both emotional expression and behavioral regulation [44,45,59]. Accordingly, recreational activities that integrate play, movement, and social interaction, such as those included in the RAP, may create emotionally supportive contexts that foster children’s motivation, persistence, and positive emotional expression during structured tasks.

Building on this framework, the present study examines changes in preschool children’s self-regulation following participation in a Recreational Activity Program (RAP) comprising structured and enjoyable recreational activities. Specifically, the program integrates playful games, rhythmic movement, and relaxation-oriented practices within a structured recreational context. Accordingly, the study investigates whether changes in children’s overall self-regulation, Attention/Impulse Control, and Positive Emotion from pretest to posttest differ between the RAP and control groups. Based on this framework, the following hypotheses were proposed:

**H1.**  *The pretest-to-posttest change in overall self-regulation will differ between the RAP and control groups, with greater improvement expected in the RAP group*.

**H2.**  *The pretest-to-posttest change in Attention/Impulse Control will differ between the RAP and control groups, with greater improvement expected in the RAP group*.

**H3.**  *The pretest-to-posttest change in Positive Emotion will differ between the RAP and control groups, with greater improvement expected in the RAP group*.

## 2. Materials and Methods

### 2.1. Study Design

This study employed a pretest–posttest control group design with classroom-level random allocation. The classroom, rather than the individual child, served as the unit of allocation, with one classroom assigned to the Recreational Activity Program (RAP) condition and one classroom assigned to the control condition. The study was reported in accordance with the CONSORT 2025 statement and the CONSORT-Children and Adolescents (CONSORT-C) 2026 extension [61,62].

### 2.2. Participants

The study sample consisted of 5–6-year-old children enrolled in preschool settings affiliated with the Ministry of National Education in the Konya district of Turkey. Among the two schools deemed eligible for participation, one participating school was selected using a lottery method. Two existing classrooms within the selected school that met the study inclusion criteria were included.

Within this school, the two existing classrooms were randomly allocated to the RAP and control conditions by an independent statistician who was unaware of the participants’ identities and characteristics, following a randomization procedure similar to that described by Erkmen Hadi et al. [63].

The primary analysis was based on participants with complete pretest and posttest outcome data who met the predefined participation criteria. For the RAP group, this required attendance at a minimum of 22 of the 24 intervention sessions. Accordingly, the primary analysis represents a complete-case, adherence-based analysis rather than an intention-to-treat analysis.

An a priori power analysis was conducted using G*Power version 3.1.9.7 [64] to estimate the minimum sample size required to detect a Group × Time interaction for the primary outcome of total self-regulation in the planned two-group pretest–posttest design. The calculation was based on the within–between interaction in a repeated-measures ANOVA, assuming an effect size of f = 0.40, a Type I error rate of 0.05, statistical power of 0.80, two groups, two measurement occasions, a correlation of 0.50 among repeated measures, and a nonsphericity correction of ε = 1. The analysis indicated an estimated minimum total sample size of 16 participants. To account for potential attrition and incomplete participation during the study, a larger sample was recruited. Initially, 21 children were allocated to the RAP group and 23 children to the control group. The final primary analysis included 38 children (19 per group), exceeding the estimated minimum sample size. The original sample size calculation was conducted at the individual-participant level and did not account for classroom-level clustering or incorporate an intraclass correlation coefficient (ICC) or design effect. Given that the study ultimately involved only two classrooms, with one classroom allocated to each condition, this represents an additional design limitation and is considered when interpreting the findings.

Initially, 21 children were allocated to the RAP group and 23 children to the control group. Two children in the RAP group did not meet the predefined attendance criterion and were therefore not included in the primary analysis. In the control group, three children were lost to follow-up because of irregular participation in the study procedures, and one additional child did not complete the posttest assessment. Consequently, the primary complete-case analysis included 19 participants in each group (*N* = 38). Participant recruitment, allocation, follow-up, and analysis are summarized in Figure 1.

The demographic characteristics of the participants included in the primary analysis are presented in Table 1.

The demographic characteristics of the participants are presented in Table 1. In the control group, 68.4% of the participants were boys and 31.6% were girls, whereas the RAP group consisted of 42.1% boys and 57.9% girls. Fisher’s exact test indicated no statistically significant difference between the groups in gender distribution (*p* = 0.191). However, statistically significant between-group differences were observed in both maternal and paternal education distributions (both *p* = 0.020). Overall, the sample was characterized by a relatively high parental educational profile, although parental education distributions differed significantly between the groups.

Children were eligible to participate if they were 5–6 years of age, were enrolled in and regularly attending one of the participating classrooms, had written informed consent provided by a parent or legal guardian, were willing to participate in the study, and had no known developmental or health condition that would interfere with participation in the activities or completion of the assessments. Children who did not meet these criteria were not included in the study.

### 2.3. Randomization

The classroom served as the unit of allocation. Among the two schools deemed eligible for the study, the participating school was selected using a lottery method. Within the selected school, two existing classrooms of 5–6-year-old children were eligible for inclusion, and both classrooms were included in the study. Classroom allocation was then determined by lottery between the two existing classrooms. The lottery was conducted by an independent statistician who had no prior knowledge of the identities or characteristics of the children in either classroom. Based on the lottery result, one classroom was assigned to the Recreational Activity Program (RAP) condition and the other to the control condition. Thus, randomization was conducted at the classroom level rather than at the individual-child level. Following classroom-level allocation, parents or legal guardians were informed about the study procedures applicable to their child’s assigned condition, and written informed consent was subsequently obtained. Participant recruitment, allocation, follow-up, and analysis are summarized in Figure 1.

### 2.4. Outcome Assessment

To minimize potential bias during outcome assessment, the researchers responsible for delivering the intervention were not involved in the pretest or posttest assessments. All pretest and posttest assessments for both the RAP and control groups were administered and scored by the same independent assessor, who was blinded to classroom allocation and was not involved in intervention delivery. The assessor administered the Preschool Self-Regulation Assessment using its standardized assessor rating form. Each target behavior was evaluated individually and scored on a 0–3 scale according to the behavior-specific scoring criteria provided for the corresponding item. The same rating form, item-level scoring criteria, and standardized assessment procedures were applied to all children at both pretest and posttest, thereby supporting consistency in outcome assessment across time points and study conditions.

Parents were informed about the purpose and procedures of the study but were not informed about classroom allocation. Blinding of the intervention provider and children was not feasible because of the nature of the behavioral intervention. Thus, blinding was limited to the outcome assessor, while parents remained unaware of classroom allocation.

### 2.5. Intervention

The RAP group participated in the Recreational Activity Program (RAP), a structured, play-based recreational intervention delivered over a 12-week period, with two sessions per week and 24 sessions in total. Each session lasted approximately 60 min and followed a three-phase structure: approximately 15 min of rhythm- and movement-based warm-up activities, 30 min of structured games, and 15 min of relaxation-oriented activities. The intervention was implemented by a researcher holding a bachelor’s degree in preschool education, with at least one additional member of the research team present during each session. Thus, a minimum of two researchers were involved in every intervention session, with three researchers present in some sessions.

Prior to implementation, the research team conducted preparatory work to support consistent delivery of the intervention according to the predefined protocol. This process included familiarization with the intervention manual, rehearsal of session content, and standardization of implementation procedures. Attendance was recorded throughout the 24-session intervention, and session-by-session implementation records were maintained to document the activities delivered and their consistency with the predefined intervention content. A separate standardized RAP-specific fidelity checklist or independent fidelity rating was not employed. Any physical problems or adverse events occurring during the intervention sessions were to be documented in the session records; no separate standardized adverse-event assessment instrument was used.

No substantive modifications were made to the overall intervention structure or core program components during implementation. However, minor activity-level adaptations were made in response to children’s engagement during sessions. For example, activities that elicited greater engagement were occasionally repeated more frequently, whereas the order of activities was adjusted when children’s attention or interest declined. These adaptations did not alter the planned session duration, three-phase structure, or core self-regulation-oriented content of the program.

The RAP was designed as a developmentally appropriate intervention aimed at supporting preschool children’s self-regulation through enjoyable and structured recreational experiences. The program was informed by theoretical and empirical evidence on structured play- and movement-based activities in relation to self-regulation and executive functioning during early childhood [34,35,49], as well as systematic reviews of multidimensional interventions incorporating play, movement, and mindfulness-related practices [54,58].

The first phase consisted of rhythm- and movement-based activities, primarily accompanied by music, to engage children’s attention, encourage active participation, and prepare them for subsequent activities. The second phase consisted of structured and rule-based games providing opportunities to practice attentional control, strategy use, imitation, communication, cooperation, self-control, patience, and turn-taking. Activities varied across sessions while maintaining the planned structure of the program. The final phase included basic yoga practices, breathing exercises, meditation, and relaxation activities intended to facilitate physiological and emotional calming. Collectively, the three-phase structure integrated physical activity, cognitive engagement, and opportunities for behavioral and emotional regulation within a structured recreational context.

### 2.6. Control Condition

Children assigned to the control condition continued their routine preschool education under the supervision of their classroom teacher throughout the 12-week study period. The routine preschool program included free play, basic movement activities, and teacher-guided educational activities delivered as part of the usual classroom schedule. The frequency and duration of these routine activities followed the regular preschool schedule and were not standardized or modified for the purposes of the study. Thus, the distinction between the conditions was not based on the complete absence of play or movement experiences in the control classroom. Rather, the RAP constituted an additional, systematically structured intervention delivered twice weekly for approximately 60 min per session over 12 weeks (24 sessions), following a predefined sequence of rhythm- and movement-based activities, structured and rule-based games, and relaxation-oriented practices. The control group did not receive this additional structured program, and its routine preschool schedule and educational activities continued without study-related modification.

RAP sessions were delivered only to the classroom allocated to the intervention condition and were not provided to the control classroom. However, possible informal exchange of intervention-related experiences between children was not independently monitored; therefore, contamination between conditions cannot be completely excluded. Following completion of the posttest assessments, children in the control group received small thank-you gifts.

### 2.7. Data Collection Tools

Personal Information Form: A brief demographic information form was developed by the researchers to collect background information about the participants. The form included questions regarding age, gender, parental education level, number of siblings, family income, and type of housing. Consistent with methodological approaches commonly used in early childhood research, these sociodemographic variables were collected as descriptive and factual information using a researcher-developed structured form [65,66].

Self-Regulation Scale: Children’s self-regulation was assessed using the Preschool Self-Regulation Assessment (PSRA), a performance-based assessment developed by Smith-Donald et al. [20]. The Turkish adaptation of the PSRA was conducted by Fındık Tanrıbuyurdu and Güler Yıldız [67]. The assessment is based on direct assessor–child interaction and includes structured tasks through which children’s attention, behavioral regulation, and emotional responses are evaluated. Each item is rated on a 4-point scale ranging from 0 to 3. The PSRA consists of 16 items and includes 10 structured tasks, such as walking in a straight line, tapping a pencil, stacking cubes, grouping objects, waiting for instructions, and delaying gratification. Factor analysis of the Turkish version identified a two-factor structure comprising Attention/Impulse Control and Positive Emotion. The Turkish version demonstrated an overall reliability coefficient of α = 0.83, with subscale reliability coefficients of α = 0.88 for Attention/Impulse Control and α = 0.80 for Positive Emotion [67]. The total PSRA self-regulation score was specified as the primary outcome of the study, while the Attention/Impulse Control and Positive Emotion subscale scores were considered secondary outcomes.

### 2.8. Data Collection

An information session was conducted for parents, teachers, and children in both the RAP and control groups to explain the study procedures and assessment schedule. Prior to data collection, written informed consent was obtained from the parents or legal guardians of all participating children. Children were also informed about the study using age-appropriate language, and their willingness to participate was respected. Participation was voluntary, and the confidentiality of participant information was maintained throughout the study.

Following classroom-level random allocation, baseline data were collected using the Personal Information Form and the Preschool Self-Regulation Assessment (PSRA). After completion of the 12-week intervention period, the PSRA was administered again to children in both groups as the posttest assessment. Each assessment session lasted approximately 20 min. All pretest and posttest assessments were conducted according to the same standardized procedures.

### 2.9. Ethical Considerations

Ethical approval for the study was obtained from the Ethics Committee of the Faculty of Sports Sciences at Selçuk University on 23 February 2023 (Approval No. 40990478-050.99-757508), prior to the commencement of the study on 27 February 2023. The study was conducted as part of a project supported by the Scientific Research Projects Coordination Unit of Selçuk University (Project No. 23401056; project start date: 3 May 2023). Written informed consent was obtained from the parents or legal guardians of all participating children prior to data collection. Children were informed about the study using age-appropriate language, and their willingness to participate was respected throughout the study. The trial was retrospectively registered on ClinicalTrials.gov (Identifier: NCT06360016; registration date: 10 April 2024; https://clinicaltrials.gov/study/NCT06360016, Accessed on 24 September 2026). Permission to use the Preschool Self-Regulation Assessment was obtained from the relevant authors.

### 2.10. Data Analysis

Categorical variables were summarized using frequencies and percentages. Continuous variables were summarized using means and standard deviations (SDs), as well as medians and interquartile ranges (IQRs), where appropriate. Distributional characteristics of the outcome variables were examined using the Shapiro–Wilk test and descriptive inspection. Given the repeated-measures design and the distributional characteristics of the outcome data, a nonparametric longitudinal approach that does not require normality of the outcome distributions was used as the primary inferential analysis.

To directly evaluate whether changes over time differed between the RAP and control groups, a nonparametric analysis of longitudinal data (nparLD) was conducted for the primary outcome of total self-regulation and the secondary outcomes of Attention/Impulse Control and Positive Emotion. Each outcome was analyzed using a two-factor repeated-measures structure, with group (RAP vs. control) as the between-subject factor and time (pretest vs. posttest) as the within-subject factor. The primary effect of interest was the Group × Time interaction, which tested whether the pretest-to-posttest pattern of change differed between the two groups. Relative treatment effects (RTEs) were also obtained to describe the distributional pattern of each outcome across group and time conditions.

As a supporting analysis, individual change scores were calculated for each outcome as posttest minus pretest, and these change scores were compared between the RAP and control groups using the Mann–Whitney U test. To provide an interpretable estimate of the magnitude and precision of the between-group difference in change, the mean change difference (RAP minus control) and its 95% bootstrap confidence interval (10,000 resamples) were additionally calculated. These analyses were used to complement, rather than replace, the primary Group × Time analysis.

Demographic characteristics were compared between groups using Fisher’s exact test because of the small and zero cell frequencies in some categories. Descriptive analyses were initially conducted using IBM SPSS Statistics version 22, whereas the revised longitudinal, change-score, bootstrap, and Fisher’s exact analyses were performed using R version 4.6.1. The nparLD package (version 2.3.1) was used for the nonparametric longitudinal analyses.

## 3. Results

This section presents the results of the analyses conducted to examine changes in self-regulation outcomes over time in the RAP and control groups. Descriptive statistics are presented first, followed by the primary nonparametric longitudinal analyses examining Group × Time interactions and supporting analyses comparing change scores between the groups. The trial was completed as planned, with no early termination or stopping. No falls, injuries, or other physical adverse events related to participation in the RAP were observed during the intervention sessions.

Descriptive statistics for the study outcomes are presented in Table 2. At pretest, the RAP group showed higher descriptive scores for attention/impulse control and total self-regulation, whereas positive emotion scores were similar across groups. From pretest to posttest, increases were observed in all three outcomes in the RAP group. Smaller increases were observed in the control group for attention/impulse control and total self-regulation, while positive emotion scores remained relatively stable. These descriptive patterns were subsequently evaluated using the nonparametric longitudinal Group × Time analysis.

To directly examine whether changes in self-regulation differed between the RAP and control groups over time, nonparametric longitudinal analyses were conducted for total self-regulation, attention/impulse control, and positive emotion. The primary effect of interest was the Group × Time interaction. Relative treatment effects (RTEs) for each group-by-time condition and the corresponding interaction test statistics are presented in Table 3.

The Group × Time interaction was statistically significant for total self-regulation (WTS = 53.47, *p* < 0.001), attention/impulse control (WTS = 42.03, *p* < 0.001), and positive emotion (WTS = 50.28, *p* < 0.001). The RTE pattern was consistent with a greater upward shift in the outcome distributions from pretest to posttest in the RAP group than in the control group across all three outcomes. Specifically, the RTE for total self-regulation increased from 0.442 to 0.869 in the RAP group, compared with 0.306 to 0.383 in the control group. Corresponding increases were observed for attention/impulse control (0.455 to 0.858 vs. 0.299 to 0.389) and positive emotion (0.389 to 0.842 vs. 0.375 to 0.394). Thus, the observed pattern of change over time differed significantly between the two groups for each outcome.

### Supporting Analysis of Change Scores

To complement the primary Group × Time analyses and quantify the magnitude of the observed changes, change scores were calculated as posttest minus pretest for each outcome. Change scores were then compared between the RAP and control groups using the Mann–Whitney U test. Mean between-group differences in change and their 95% bootstrap confidence intervals were additionally calculated to provide estimates of the magnitude and precision of the observed differences. Rank-biserial correlations (*r*_rb_) were also calculated as effect-size estimates for the between-group change-score comparisons, with 95% bootstrap confidence intervals used to quantify their precision (Table 4).

As shown in Table 4, the RAP group demonstrated a greater mean increase in total self-regulation (17.53 ± 5.64) than the control group (3.26 ± 3.74), corresponding to a mean between-group difference in change of 14.26 points (95% bootstrap CI [11.37, 17.21]; W = 357.0, *p* < 0.001). Greater increases were also observed in the RAP group for attention/impulse control (10.68 ± 4.67 vs. 3.00 ± 3.61; mean difference = 7.68, 95% bootstrap CI [5.16, 10.26]; W = 332.5, *p* < 0.001) and positive emotion (6.84 ± 3.17 vs. 0.32 ± 1.73; mean difference = 6.53, 95% bootstrap CI [4.89, 8.05]; W = 351.0, *p* < 0.001). These supporting analyses were consistent with the primary longitudinal findings, showing greater observed pretest-to-posttest increases in the RAP group across all three outcomes.

## 4. Discussion

The present study examined whether changes in self-regulation over time differed between preschool children participating in the Recreational Activity Program (RAP) and those receiving routine preschool education. The primary nonparametric longitudinal analyses showed significant Group × Time interactions for total self-regulation, Attention/Impulse Control, and Positive Emotion, indicating that the pattern of change from pretest to posttest differed between the two groups. Across all three outcomes, the RAP group showed greater observed increases than the control group. Supporting change-score analyses were consistent with these longitudinal findings, with between-group differences in change favoring the RAP group and bootstrap confidence intervals excluding zero. Taken together, these findings suggest that participation in the structured, multicomponent RAP was associated with greater short-term improvements in the assessed self-regulation outcomes compared with routine preschool education. However, these findings should be interpreted cautiously in light of the small sample, baseline differences, and classroom-level allocation with only one classroom per study condition.

At baseline, no statistically significant between-group difference was observed in total self-regulation scores (*p* = 0.090), although the RAP group showed descriptively higher scores. A statistically significant baseline difference was observed for Attention/Impulse Control (*p* = 0.043), indicating that complete baseline equivalence was not achieved for this subscale. This imbalance is particularly important given the small sample size and should be considered when interpreting the findings. Although the primary longitudinal analysis directly evaluated whether patterns of change over time differed between the groups, the baseline imbalance limits the extent to which the observed differences in change can be attributed specifically to the RAP. Accordingly, the findings for Attention/Impulse Control should be interpreted with particular caution. It is also noteworthy that the control group showed smaller increases in Attention/Impulse Control and total self-regulation over time, while Positive Emotion remained relatively stable. These changes may partly reflect natural developmental progression during early childhood as well as children’s continued exposure to routine classroom activities. Given that self-regulatory capacities continue to develop during the preschool years, some change over time would be expected. Nevertheless, the significantly different Group × Time patterns, together with the greater observed increases in the RAP group, suggest that participation in the structured RAP was associated with additional short-term changes beyond those observed under routine preschool education.

The observed changes in self-regulation can be considered in relation to the components included in the RAP intervention. One possible interpretation of the greater observed improvement in Attention/Impulse Control in the RAP group concerns the structured and rule-based games and relaxation-oriented practices, such as basic yoga and meditation, incorporated into the multicomponent program. Rule-based games require children to follow instructions, inhibit impulsive responses, and sustain attention, and may therefore provide opportunities to practice cognitive control processes [19,68]. Similarly, mindfulness-based activities, including simple breathing exercises and relaxation practices, have been suggested to support the regulation of arousal, sustained attention, and behavioral control during tasks [69]. These potential mechanisms are consistent with previous studies reporting beneficial associations of structured games and mindfulness-based practices with attention regulation and inhibitory skills in early childhood [55,70]. However, because these components were delivered together as part of a multicomponent program, the present study cannot determine the extent to which any individual RAP component contributed to the observed changes.

The greater observed improvement in Positive Emotion may similarly be considered in relation to the emotionally engaging and enjoyable nature of the RAP activities. Rhythmic games, music-based movement activities, and socially interactive play may foster enjoyment, enthusiasm, and active engagement, which are relevant indicators of positive emotional expression in early childhood contexts [55,71]. From a theoretical perspective, recreational environments emphasizing enjoyment and intrinsic motivation may enhance emotional engagement and participation [12]. Accordingly, the combination of playful interaction, rhythmic movement, and social engagement within the RAP may have provided an emotionally supportive context for positive affect and sustained involvement in goal-directed activities. This interpretation is consistent with evidence indicating that preschool children’s positive engagement with teachers, peers, and classroom tasks is associated with developing self-regulatory capacities [72,73]. Nevertheless, Positive Emotion as assessed in the present study primarily reflects observable positive emotional expression and engagement during the assessment context and should not be interpreted as a direct measure of emotion regulation.

The structured and sequential nature of the RAP, together with repeated opportunities for active participation and practice, is also consistent with characteristics identified as relevant to school-based self-regulation interventions [74]. Longitudinal evidence indicates that the preschool years represent an important developmental period for behavioral self-regulation, with substantial development occurring between approximately three and seven years of age and considerable variation in developmental trajectories according to individual and environmental characteristics. In this context, structured recreational experiences during the preschool years may provide opportunities to practice self-regulatory behaviors within developmentally appropriate activity settings.

More broadly, previous intervention research has reported favorable developmental outcomes from approaches targeting self-regulation in early childhood. Liu [75], for example, identified physical activity, play-based practices, and mindfulness applications as potentially useful approaches for supporting self-regulation in young children. Rhythm-based and auditory cue interventions have also been associated with improvements in regulatory processes [76,77], providing a relevant context for the rhythm-based activities incorporated into the RAP. The role of play and recreational engagement is further supported by longitudinal evidence; Colliver et al. [8] reported that time spent in unstructured play predicted children’s later self-regulation skills. Collectively, this literature suggests that both structured and unstructured activity environments may provide meaningful opportunities for practicing regulatory skills during early childhood.

Regarding Attention/Impulse Control, previous research has reported beneficial findings for play- and movement-based interventions [78,79]. The pattern observed in the present study is consistent with findings from rhythm- and movement-based interventions [55,71], outdoor activity programs [80], and body-awareness-based play [79]. Similarly, the ENGAGE program, which combines play and physical activity, has been associated with improvements in cognitive control and attention-related outcomes in children [81]. Rather than demonstrating an effect of any particular RAP component, the present findings are therefore consistent with the broader literature suggesting that structured play and movement-based experiences may provide contexts in which attentional and behavioral regulation can be practiced.

With respect to Positive Emotion, the present findings can be interpreted alongside literature emphasizing associations between positive emotional experiences, engagement, and self-regulatory processes [82,83]. Play-based experiences are often characterized by enjoyment and engagement, which may support children’s motivation and involvement in goal-directed activities. Previous research has shown that positive emotions such as enjoyment, hope, and pride are associated with self-regulation [84,85] and may facilitate motivation, cognitive flexibility, and task engagement [86]. Conversely, self-regulatory capacities may themselves contribute to more positive emotional experiences, particularly in achievement contexts [87], highlighting the potentially reciprocal nature of these associations. Although evidence in preschool populations remains more limited, Li et al. [88] reported associations between positive emotions and multiple components of self-regulation. In the present study, the observed change in Positive Emotion may therefore reflect greater positive emotional expression and engagement within the assessment context following participation in the RAP. Opportunities for enjoyment and social interaction in recreational settings may provide supportive contexts for children’s emotional engagement and broader developmental experiences [44,89,90]. However, the present findings do not establish that the RAP improved emotion regulation itself.

The characteristics of the sample should also be considered when interpreting the findings. Participants were characterized by a relatively high parental educational profile, and maternal and paternal education distributions differed between the RAP and control groups. These characteristics may limit the extent to which the findings can be generalized to preschool populations with more diverse family backgrounds. Previous research has also indicated that family and contextual factors may be associated with variation in intervention outcomes [55]. Future studies involving more diverse samples would therefore be valuable for examining whether similar patterns are observed across different educational and family contexts.

Finally, the present findings reflect short-term changes observed immediately following the 12-week intervention. Previous research suggests that play-based, movement-focused, and mindfulness-based interventions may be associated with changes in attention, behavioral regulation, and related developmental outcomes [57,91,92]. However, short-term changes do not establish that such differences will persist over time. Longer-term follow-up is therefore required to determine whether the observed patterns are maintained and whether they are associated with subsequent developmental outcomes [93,94].

Several limitations should be considered when interpreting the present findings. Most importantly, classroom-level allocation involved only two existing classrooms, with one classroom assigned to the RAP condition and one to the control condition. Consequently, the study condition was completely confounded with classroom membership, and potential influences associated with teacher characteristics, classroom environment, peer context, or other classroom-level factors cannot be separated from the observed between-group differences. The study therefore does not provide replicated classroom-level evidence of an intervention effect, and the findings should be regarded as preliminary. Future studies should include multiple classrooms within each condition and employ analytical approaches that appropriately account for clustering.

A second limitation concerns baseline differences between the groups. Although no statistically significant baseline difference was observed for total self-regulation, the groups differed significantly in Attention/Impulse Control. In addition, both maternal and paternal education distributions differed significantly between the groups. Given the small sample size, these baseline imbalances may have contributed to the observed patterns of change and should be considered when interpreting the findings, particularly those concerning Attention/Impulse Control. The relatively small sample and the high parental educational profile of the participants also limit the generalizability of the findings to broader preschool populations with more diverse family backgrounds.

The multicomponent nature of the RAP represents an additional interpretive limitation. Rhythm- and movement-based activities, structured games, and relaxation-oriented practices were delivered as components of a single integrated program. Therefore, the present design does not permit the relative contribution of individual program components to the observed changes to be determined. Although attendance and session-by-session implementation records were maintained to document delivery of the planned intervention, the study did not include a standardized RAP-specific fidelity measure or independent fidelity assessment. Future studies could incorporate systematic fidelity evaluation and component-specific designs to examine both consistency of implementation and the potential contribution of individual program elements.

Finally, outcomes were assessed immediately following the 12-week intervention, and no longer-term follow-up assessment was conducted. It therefore remains unknown whether the observed changes were maintained beyond the intervention period. Although outcome assessments were conducted by the same independent assessor who was blinded to classroom allocation and used standardized assessment and item-level scoring procedures, the nature of the behavioral intervention precluded blinding of the intervention providers and participating children. Future research involving larger and more diverse samples, multiple classrooms within each condition, longer-term follow-up, and replicated classroom-level allocation is needed to provide more robust evidence regarding the potential role of structured recreational programs in supporting self-regulation during early childhood.

## 5. Conclusions

The present study provides preliminary evidence that participation in a structured, multicomponent Recreational Activity Program (RAP) was associated with greater short-term improvements in self-regulation among preschool children compared with routine preschool education. Significant Group × Time interactions were observed for total self-regulation, Attention/Impulse Control, and Positive Emotion, indicating different patterns of change over time between the RAP and control groups.

However, the findings should be interpreted cautiously. In particular, the inclusion of only one classroom per study condition prevents the observed differences from being separated from potential classroom- or teacher-level influences, while baseline differences between the groups further limit causal interpretation. Accordingly, the present findings should be regarded as preliminary and require confirmation in larger studies involving multiple independent classrooms within each condition.

## Figures and Tables

**Figure 1:**
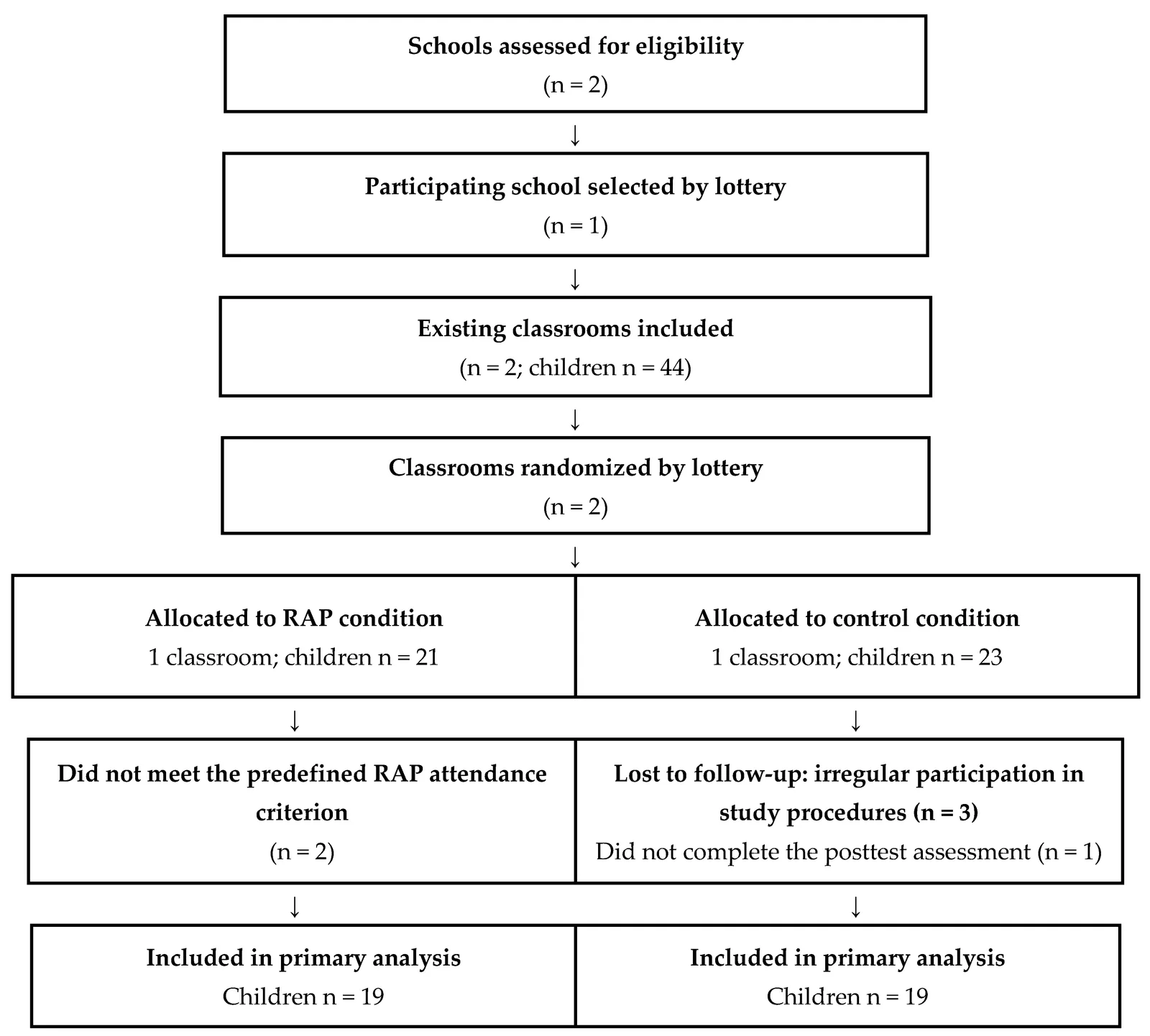
Participant and classroom flow through the study.

**Table 1:** Demographic Characteristics of Participants by Group.

Variable	Category	Control *n* (%)	RAP *n* (%)	Fisher’s Exact *p*
Gender	Female	6 (31.6)	11 (57.9)	0.191
	Male	13 (68.4)	8 (42.1)	
Mother’s education	Bachelor’s degree	0 (0.0)	6 (31.6)	0.020
	Postgraduate degree	19 (100.0)	13 (68.4)	
Father’s education	Bachelor’s degree	0 (0.0)	6 (31.6)	0.020
	Postgraduate degree	19 (100.0)	13 (68.4)	

Note. RAP = Recreational Activity Program. Fisher’s exact test was used because of small and zero cell frequencies.

**Table 2:** Descriptive Statistics for Self-Regulation Outcomes by Group and Time.

Outcome	Group	Pretest M ± SD	Pretest Median (IQR)	Posttest M ± SD	Posttest Median (IQR)
Attention/Impulse Control	RAP	15.74 ± 4.86	17.00 (4.00)	26.42 ± 2.95	27.00 (3.00)
	Control	11.37 ± 6.35	10.00 (11.00)	14.37 ± 5.66	14.00 (7.00)
Positive Emotion	RAP	7.32 ± 2.36	8.00 (2.50)	14.16 ± 3.13	14.00 (5.50)
	Control	7.32 ± 2.26	7.00 (1.50)	7.63 ± 2.50	7.00 (3.50)
Total Self-Regulation	RAP	23.05 ± 6.06	24.00 (7.00)	40.58 ± 4.26	40.00 (5.50)
	Control	18.74 ± 7.25	19.00 (12.00)	22.00 ± 7.00	22.00 (6.50)

Note. RAP = Recreational Activity Program; M = mean; SD = standard deviation; IQR = interquartile range.

**Table 3:** Nonparametric longitudinal analysis of self-regulation outcomes.

Outcome	RAP Pretest RTE	RAP Posttest RTE	Control Pretest RTE	Control Posttest RTE	Group × Time Statistic WTS	*p*
Total Self-Regulation	0.442	0.869	0.306	0.383	53.47	<0.001
Attention/Impulse Control	0.455	0.858	0.299	0.389	42.03	<0.001
Positive Emotion	0.389	0.842	0.375	0.394	50.28	<0.001

Note. RTE = relative treatment effect; RAP = Recreational Activity Program; WTS = Wald-type statistic. The Group × Time effect tests whether the pattern of change from pretest to posttest differed between the RAP and control groups. RTE values range from 0 to 1, with higher values indicating relatively higher outcome distributions.

**Table 4:** Between-Group Comparison of Change Scores in Self-Regulation Outcomes.

Outcome	RAP Change M ± SD	Control Change M ± SD	W	Mean Difference	95% Bootstrap CI	Rank-Biserial *r* (95% Bootstrap CI)	*p*
Total Self-Regulation	17.53 ± 5.64	3.26 ± 3.74	357.0	14.26	[11.37, 17.21]	0.978 [0.911, 1.000]	<0.001
Attention/Impulse Control	10.68 ± 4.67	3.00 ± 3.61	332.5	7.68	[5.16, 10.26]	0.842 [0.632, 0.986]	<0.001
Positive Emotion	6.84 ± 3.17	0.32 ± 1.73	351.0	6.53	[4.89, 8.05]	0.945 [0.845, 1.000]	<0.001

Note. Change scores were calculated as posttest minus pretest. Positive values indicate increases from pretest to posttest. Mean differences were calculated as RAP minus control. The 95% confidence intervals for the mean differences were estimated using percentile bootstrap resampling with 10,000 resamples. Between-group differences in change scores were evaluated using the Mann–Whitney U test. Rank-biserial correlation (*r*_rb_) was used as the effect-size measure for the between-group change-score comparisons; its 95% confidence intervals were estimated using 5000 bootstrap resamples. Positive *r*_rb_ values indicate greater change in the RAP group. RAP = Recreational Activity Program; CI = confidence interval.

## Data Availability

The datasets generated during and/or analyzed during the current study are available from the corresponding author on request.

## Abbreviations

The following abbreviations are used in this manuscript:

RAP	Recreational Activity Program
